# Mutation analysis in exons 22 and 24 of SCN4A gene in Iranian patients with non-dystrophic myotonia

**Published:** 2015-10-07

**Authors:** Mohammad Mehdi Heidari, Mehri Khatami, Shahriar Nafissi, Faezeh Hesami-Zokai, Afshin Khorrami

**Affiliations:** 1 Department of Biology, School of Science, Yazd University, Yazd, Iran; 2 Department of Neurology, School of Medicine, Tehran University of Medical Sciences, Tehran, Iran

**Keywords:** Nondystrophic Myotonia, Mutation, SCN4A, Polymerase Chain Reaction-Single Strand Conformational Polymorphism

## Abstract

**Background:** Non-dystrophic myotonias are a heterogeneous set of skeletal, muscular channelopathies, which have been associated with point mutations within sodium channel α-subunit (SCN4A) gene. Because exons 22 and 24 of SCN4A gene are recognized as hot spots for this disease, the purpose of the study is to identify mutation in exons 22 and 24 of SCN4A gene in Iranian non-dystrophic myotonias patients.

**Methods: **In this study, 28 Iranian patients with non-dystrophic myotonia analyzed for the mutation scanning in exons 22 and 24 of SCN4A gene by polymerase chain reaction-single strand conformational polymorphism (PCR-SSCP) and sequencing.

**Results: **We found 29073G>C substitution in SCN4A gene in one case and 31506A>G substitution in seven cases. The 29073G>C substitution causes a missense mutation G1306A, located in the conserved cytoplasmic loop connecting repeat III and IV of the SCN4A channel but, 31506A>G substitution do not alter amino acid in SCN4A protein.

**Conclusion:** G1306A residue is located in functionally important protein region. In “hinged-lid model” for Na^+^ channel inactivation in which glycines^1306^ act as the hinge of the lid occluding the channel pore. Mutation in this region slowed fast inactivation. Therefore, it might be a pathogenic mutation. The causal relationship of this mutation with the disease is an object for further discussion.

## Introduction

Muscle channelopathies (the inherited muscle ion channel diseases) are rare disorders of the skeletal muscle. Non-dystrophic myotonias are a heterogeneous set of skeletal, muscular channelopathies, which have been associated with specific point mutations within sodium channel α-subunit (SCN4A) or Cl–channel (CLCN1) genes.^1^^,^^2^ The prevalence of non-dystrophic myotonia has been estimated to be ~1 in 100,000 in the worldwide.^   3 ^ Voltage-gated sodium channels are prominent transmembrane proteins in excitable tissues and are responsible for the rising phase of the action potential in the membranes of neurons and most electronically excitable cells.^1^^,^^4^ 

The skeletal muscle sodium channel comprises a principal pore-forming and voltage sensing subunit (the alpha subunit), which is associated with an accessory beta-1 subunit. The beta-1 subunit has not been reported to be linked to any disease. It’s alpha is encoded by the SCN4A gene, which is located on chromosome 17q23-25, comprises 24 exons with a 5.5 kb open reading frame, is associated with various neuromuscular disorders.^   5 ^ The alpha subunit consists of four homologous domains, and each domain possesses six hydrophobic putative transmembrane segments (S1–S6).^   4 ^ Conserved sequences in these channels promote specific functions.^   6 ^^,^^   7 ^

SCN4A mutations produce several clinically distinct skeletal muscle disorders including hyperkalemic periodic paralysis, paramyotonia congenita, potassium-aggravated myotonia, hypokalemic periodic paralysis, and congenital myasthenic syndrome.^   5 ^

The similarities between sodium channel myotonia and myotonia congenita can lead to difficulty in prioritizing genetic testing. Clinical history and examination considered in conjunction with electromyogram findings can improve the ability to distinguish between the two and guide genetic analysis, but in some cases screening of SCN4A genes will be required.^   8 ^

More than 40 mutations have been reported in SCN4A gene, but exons 22 and 24 of SCN4A gene are recognized as hot spots for myothonia,^   9 ^ and there is no study investigating on Iranian patients with non-dystrophic myotonia, so the aim of this study was to screen this hotspot exon of SCN4A gene in Iranian patients with non-dystrophic myotonia by polymerase chain reaction-single strand conformational polymorphism (PCR-SSCP) and sequencing.

## Materials and Methods

Twenty-eight Iranian patients with non-dystrophic myotonia were included in the present study (Table 1). The control group comprised 30 healthy controls that matched for age, sex, and ethnicity. Control subjects had no signs of the neuromuscular disease when enrolled in the study. All of the patients and the control group were informed of the aims of the study and gave their informed consents for the genetic analysis. Patients were referred for assessment by consultant neurologists in Iran.

DNA was obtained directly from peripheral blood samples by chloroform extraction and ethanol precipitation. Samples of genomic DNA were amplified by the polymerase chain reaction (PCR) with specific primers. The experimental conditions were optimized for each pair of primers.

The following primer pairs were designed to amplify the exon 22 of the SCN4A gene and the exon 24 of SCN4A gene (Table 2). Primers were designed by Primer Design Software (Primer Premier 5.0; Premier Biosoft Inc., Canada), and their secondary structure was examined with Gene Runner version 3.05 (Hastings Software Inc. Hastings, NY, USA, http://www.generunner.com).

PCR was performed in a total volume of 25 µl containing 100 ng of template DNA, 10 pmol of each primer and 1× PCR Master Mix (Yekta Tajhiz Azma, Tehran, Iran). The PCR was performed based on the following conditions: initial denaturation at 94° C for 2 minutes; followed by 35 cycles including denaturation at 94 °C for 35 seconds, annealing at 64 °C (exon 22) and 62 °C (exon 24) for 50 seconds, and extension at 72 °C for 30 seconds; and a final extension at 72 °C for 5 minutes followed by a final extension for 5 minutes. The PCR products were electrophoresed on an ethidium bromide-stained 2% agarose gel.

The amplified PCR products were analyzed using SSCP analysis.   10  6 µl of the amplified samples were diluted with 6 µl of SSCP loading buffer dye, denatured at 94° C for 3 minutes and then kept on ice for 5 minutes until loaded onto 8% polyacrylamide gels. Gels were run at 120 V for 12 hours in a buffer containing TBE ×0.5 (pH = 8.3). After electrophoresis; the gels were stained by silver nitrate.

**Table 1:** Clinical data for non-dystrophic myotonia patients

**Group**	**Sex**	**Number**	**Age**	**Age of onset**	**Effected of family**
Patient	Male	14	36 ± 12	9 ± 5	16 famillial
	Female	14			12 sporadic
Healthy control	Male	17	34 ± 14	-	-
	Female	13			

**Table 2:** Primers used for amplification of sodium channel α-subunit (SCN4A) gene

**Exon**	**Primer sequence (5'-3')**	**Temperature (°C)**	**Size (bp)**
22	F: TGGAGGCAGGAAGGGGAACT R: GGCAGCACACACAGGACAGG	64	197
24	F: TTCGAGACCTTCGGCAACAG R: CGGCTGTAGGCGATGAACTG	62	362

F: Forward; R: Reverse

**Figure 1:**
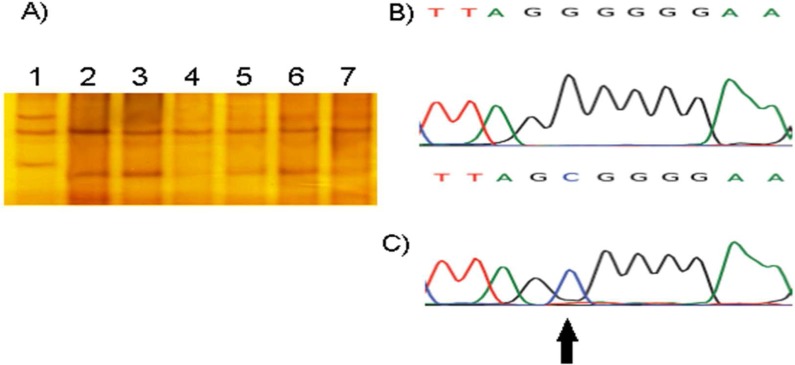
(A) Single strand conformational polymorphism gel electrophoresis of exon 22 of the SCN4A gene. Line 1 shows different pattern banding regard to lines 2, 3, 4, 5, 6, and 7. Line 7 is normal control. (B) Chromatogram of sample 7 (without mutation) and (C) Chromatogram of sample 1 (without 29073G>C mutation).

**Figure 2:**
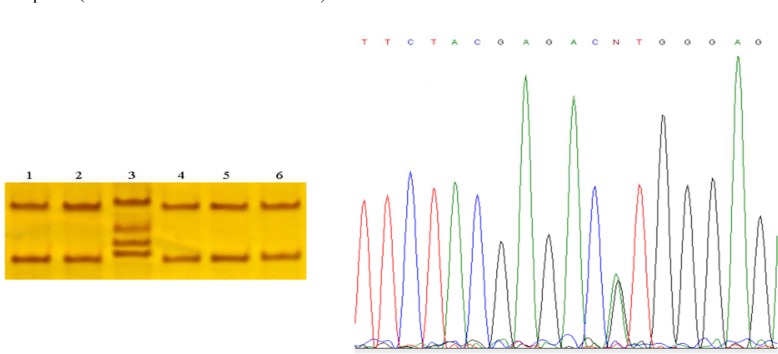
Single-strand conformational polymorphism results. Line 3 shows shift bands in a patient with a heterozygous variation. Lines 1, 2, 4, and 5 show patients without any variations and line 6 is a control sample. Sequence analysis of the genomic DNA of the patient revealed the heterozygous transition of an A to G at position 31506 in exon 24 SCN4A genes in a patient with non-dystrophic myotonia (left)

PCR products of samples with various band patterns in SSCP gel were sent to a commercial agency (Macrogene Seoul, South Korea) for sequencing. The online multiple sequence alignment software; ClustalW2 (http://www.ebi.ac.uk/ tools/msa/ clustalw2/) and Blast analysis was used to find the percent homology of the sequences that has been obtained in the study and with all other sequences of the other species.

Levels of the quantitative variables are presented as a mean ± standard deviation. Student’s t-test was used for comparison of continuous variables; Fisher’s exact test was used for comparison of categorical variables. The GraphPad Prism software (version 3.00, GraphPad Software, La Jolla, CA, USA) was used for statistical analysis, with P < 0.05 considered indicative of statistical significance.

## Results

The mobility of single-stranded DNA fragments in SSCP gel was conducted on 28 patients and 30 healthy controls. Mean age was 36 ± 12 and 34 ± 14 years for patients and controls, respectively. The screening of exon 22 of SCN4A gene led to the identification of one mutation in one out of 28 patients. DNA sequencing revealed 29073G>C variant (Figure 1). This variant causes a missense mutation G1306A (Glycine to Alanine).

The SSCP and DNA sequencing of exon 24 revealed a synonymous heterozygous 31506A>G variation in seven out of 28 patients. This variation does not change an amino acid in SCN4A protein and has not been previously reported in SCN4A gene (Figure 2). 

**Figure 3:**
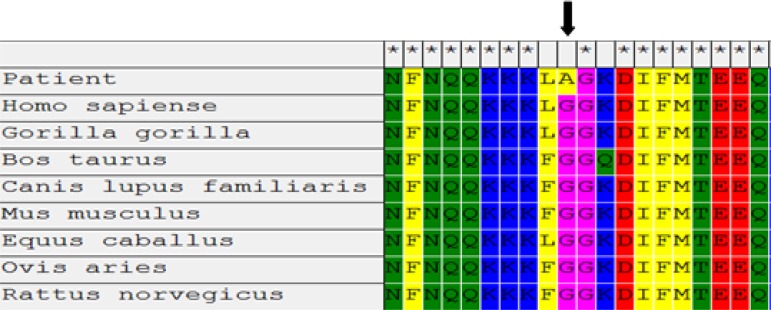
Multiple sequence alignment of a part of exon 22 from various species. Glycine 1306 is completely conserved among all Na^+ ^channel α-subunits

The results of multiple sequence alignment with various species showed that G1306 is conserved during evolution (Figure 3).

## Discussion

The clinical signs and electrophysiological indicators are used to prioritize genetic testing in the non-dystrophic myotonias but, the detection of sodium channel myotonia from dominant myotonia congenital is difficult. Hence, it requires that a proportion of patients to the screen of both SCN4A and CLCN-1 genes.   11  Lerche et al. found three point mutations at the same nucleotide position 29073 of the SCN4A gene in three families with a form of myotonia. These mutations change glycine 1306 to glutamic acid, valine, or alanine in SCN4A protein.^        12 ^

Furthermore, McClatchey et al. found one of the three substitutions at location 1306 (glycine-to-valine) in a family characterized by chronic myotonia.^   13 ^ Vicart et al. demonstrated that the most common sodium channel myotonia mutations are V1589M and G1306 position.^   14 ^

Matthews et al. studied the clinical and genetic features a long cohort of UK patients with non-dystrophic myotonia and they demonstrated that 3 of their patients had mutations (G1306A, G1306E) that previously described and identified two novel mutations (R1448L, L1436P) in SCN4A gene.^   9 ^

Here, we found one homozygous missense mutation G1306A (Glycine to Alanine) in SCN4A gene in one patient with mild painful myotonia. Multiple sequence alignment with various species showed that G1306 is conserved during evolution (Figure 3). Data of Polyphen-2 software (with 0.54 score) predicted this mutation is a possible pathogen. This substitution has been previously predicted in the conserved cytoplasmic loop connecting repeat III and IV domains of the sodium channel α-subunit could cause to inactivation of gate of the sodium channel. The glycine 1306 confers a good flexibility of the hinge that could restricted by side-chains of other amino acids.^12^^,^^15^^,^^16^ 

Second nucleotide variation, the heteroplasmic 31506 A>G polymorphism in exon 24 SCN4A gene, which no alter amino acid sequences, has not been previously described. This synonymous mutation is located in a moderately conserved amino acid of the C-terminal loop of SCN4A protein.

***Study limitations***

No accessibility to tissue samples from our patients is the major limitation of our study. Another limitation is the lack of classification of the patients according to their clinical findings. Further studies with larger cohorts of patients are warranted to reveal the relationship of these nucleotide changes with non-dystrophic myotonias.

## Conclusion

Our mutational analysis confirms the role of single nucleotide polymorphisms in SCN4A gene in Iranian patients with non-dystrophic myotonias. Hence, to find out and understand the nature of pathogenesis and predisposition effects of these variations on non-dystrophic myotonias, further genetic, and functional studies are necessary.

## Conflict of Interests

The authors declare no conflict of interest in this study.

## Notes:

**How to cite this article: **Heidari MM, Khatami M, Nafissi Sh, Hesami-Zokai F, Khorrami A. Mutation analysis in exons 22 and 24 of SCN4A gene in Iranian patients with non-dystrophic myotonia. Iran J Neurol 2015; 14(4): 190-4.
